# P02 Strategies for Older People living in care homes to prevent Urinary Tract Infection: the ‘StOP UTI’ realist synthesis of evidence

**DOI:** 10.1093/jacamr/dlad077.005

**Published:** 2023-08-02

**Authors:** Jacqui Prieto, Jennie Wilson, Alison Tingle, Emily Cooper, Melanie Handley, Jo Rycroft-Malone, Jennifer Bostock, Heather Loveday

**Affiliations:** School of Health Sciences, University of Southampton, University Road, Southampton SO17 1BJ, UK; Richard Wells Research Centre, University of West London, St Mary’s Road, London W5 5RF, UK; Richard Wells Research Centre, University of West London, St Mary’s Road, London W5 5RF, UK; Primary Care and Interventions Unit, HCAI, Fungal, AMR, AMU & Sepsis Division, UK Health Security Agency, UK; Centre for Public Health and Community Care, University of Hertfordshire, De Havilland Campus, Mosquito Way, Hatfield AL10 9EU, UK; Faculty of Health and Medicine, Lancaster University, Bailrigg, Lancaster LA1 4YW, UK; Patient and Public Involvement, UK; Richard Wells Research Centre, University of West London, St Mary’s Road, London W5 5RF, UK

## Abstract

**Background:**

Urinary tract infection (UTI) is the most diagnosed infection in older people living in care homes, accounting for more than 50% of antibiotic prescriptions in this setting. Older people often present with non-specific or atypical symptoms, which may have other origins and are difficult for care home staff to interpret.

**Objectives:**

To identify interventions that could be effective for preventing and recognizing UTI in older people living in care homes in the UK and explore the mechanisms by which they work, for whom and under what circumstances.

**Methods:**

A synthesis of evidence using a realist approach was undertaken to develop, test and refine programme theories, which are the units of analysis within the realist approach. These were expressed as context + mechanism=outcome configurations (CMOc). Their practical relevance and potential for implementation was established through consultation with stakeholders and teacher-learner interviews.

**Results:**

We identified nine CMOc, which describe what needs to happen in care homes to facilitate improvement in practice for the prevention and recognition of UTI. These were arranged under three theory areas: (i) supporting accurate recognition of UTI; (ii) preventing UTI and catheter-associated UTI; and (iii) the infrastructure and systems required to make best practice happen. Our programme theories draw on evidence from a range of areas including infection prevention and control, antimicrobial stewardship, leadership and safety culture in care homes and person-centred care. The findings suggest a whole care team approach, involving residents, their family carers, care home staff and visiting health professionals is needed to develop and implement strategies to improve UTI prevention and recognition. Support at system level, with regulatory and inspection frameworks aligned to evidence on prevention and recognition of UTI, is imperative to ensure the resources and infrastructure are available to enable care home managers and their staff to prioritize this as part of person-centred care.

**Conclusions:**

Our findings have identified the active components of strategies that are effective in preventing and recognizing UTI in older people living in care homes and will help guide delivery of future improvement programmes and research.

